# Gender differences in the use of atypical antipsychotic medications for ICU delirium

**DOI:** 10.1186/s13054-018-2143-5

**Published:** 2018-09-21

**Authors:** Kunal Karamchandani, Robert S. Schoaps, Jillian Printz, Jeffrey M. Kowaleski, Zyad J. Carr

**Affiliations:** 1 Department of Anesthesiology & Perioperative Medicine, Penn State Health Milton S. Hershey Medical Center, 500 University Drive, Hershey, PA 17033 USA; 2 Department of Medicine, Section of Palliative Care, Penn State Health Milton S. Hershey Medical Center, Hershey, PA USA

Intensive care unit (ICU) delirium, an acute fluctuating disturbance of cognition associated with critical illness, is associated with increased mortality, ICU length of stay, mechanical ventilation, and hospital costs [1–3]. Despite a link to increased long-term mortality, atypical antipsychotic medications (AAP) are frequently administered for the treatment of ICU delirium. Males have a higher risk of being diagnosed with ICU delirium (63% vs 36%) and being initiated on AAP (44% vs 40%) compared to females [4, 5].

In this retrospective investigation, we hypothesized that male gender was more likely to be associated with hyperactive symptoms of ICU delirium, and that males were more likely to be discharged on AAP after an ICU stay. After obtaining approval from the Institutional Review Board, we performed a retrospective analysis on patients admitted to the adult ICUs at the Penn State Health Milton S. Hershey Medical Center between January 2012 and December 2014. Charts were reviewed for the following inclusion criteria: age older than 18 years and AAP initiation in the ICU. Patients were excluded if they were on AAP prior to ICU admission. Documentation was analyzed for symptoms associated with AAP initiation based on the previously described Intensive Care Delirium Checklist Worksheet (ICDSC), pre-existing psychiatric diagnoses, and ICU type. Analyses were performed using SAS (v. 9.4; SAS, NC, USA) and significance was set at *p* < 0.05.

Of 12,984 patients admitted between 2012 and 2014, 346 (2.6%) patients were newly initiated on an AAP during their ICU stay, and 32 (8.6%) patients expired prior to discharge. In total, 346 patients and 314 patients were analyzed for initial and discharge-related variables, respectively (Table 1). No gender differences were observed in the concurrent psychiatric diagnoses of major depression (*p* = 0.13), bipolar disorder (*p* = 0.54), or schizophrenia (*p* = 0.99). However, males had a higher length of ICU stay compared to females (*p* = 0.002) but not total hospital stay (*p* = 0.07). As previously observed, males had higher rates of initiation of AAP (*p* = 0.0001) and continuation after discharge (*p* = 0.034). We demonstrated that males were more likely to have documentation of agitation (*p* = 0.032), hallucinations (*p* = 0.018), impulsiveness (*p* = 0.033), and combativeness (*p* = 0.001) compared to females. No differences were found in documentation of restlessness (*p* = 0.251), confusion (*p* = 0.60), insomnia (*p* = 0.70), lethargy (*p* = 0.34), or depressed affect (*p* = 0.62).

**Table 1:** Demographics and clinical characteristics of ICU patients initiated on AAP for delirium (*N* = 346)

Characteristic	Male (*n* = 230)	Female (*n* = 116)	*p* value*
Age (years), mean (± SD)	60.1 (± 19.3)	58.4 (± 21.0)	0.459
Psychiatric diagnoses, *n*			
Major depression	82	51	0.133
Bipolar depression	18	7	0.543
Schizophrenia	6	3	0.990
Delirium manifestations^b^			
Hyperactive symptoms, *n*			
Agitation	213	99	0.032*
Hallucination	159	94	0.018*
Combativeness	139	90	0.001*
Impulsiveness	121	75	0.033*
Restlessness	58	36	0.251
Hypoactive symptoms, *n*			
Confusion	26	11	0.605
Insomnia/altered sleep	169	83	0.704
Lethargy	56	23	0.344
Depressed affect	35	20	0.627
ICU subtype, *n* (%)			
Medical	53 (23)	36 (31)	0.201
Surgical^c^	177 (77)	80 (69)	
Length of stay (days), median (IQR)			
ICU	11.0 (1–21)	8 (1–15)	0.002*
Hospital	18 (0.25–35.75)	16 (0.75–31.25)	0.070
Discharge disposition, *n* (%)			
Home	37 (16)	37 (18.1)	0.548
Expired	24 (10.4)	8 (6.8)	
Long-term care facility	163 (72.6)	86 (74.1)	
Continuation of AAP on discharge, *n* (%)	123 (70.7)	51 (29.3)	0.034*

*AAP* atypical antipsychotic medications, *ICU* intensive care unit, *IQR* interquartile range, *SD* standard deviation

^a^*p* values from two-sample *t* test (mean), chi-square test (*n*), or Mann–Whitney test (median and interquartile range)

^b^*p* value from chi-square test, χ^2^ critical value = 3.841, *df* = 1, α = 0.05

^c^Aggregated from neurosurgical, surgical, trauma, and cardiothoracic ICU populations. Medical implies all others

*Significant at *p* < 0.05

Prior studies have demonstrated higher rates of AAP initiation and continuation in male ICU patients. To our knowledge, our investigation is the first to show an association between male gender symptoms of hyperactive delirium and initiation of AAP in the ICU. As hyperactive symptoms are more visible and more likely to invoke safety concerns, we suspect that this leads to a higher rate of initiation and continuation of AAP in male patients. Thus, hyperactive symptoms drive the gender differences observed in AAP administration in the ICU. Further research is required to substantiate these findings and assess their clinical implications.

## Abbreviations

AAP: Atypical antipsychotic medications
ICU: Intensive care unit
